# Treatment of a Woman with Inoperable Meningioma Using Mifepristone for 26 Years

**DOI:** 10.1155/2020/5162918

**Published:** 2020-02-11

**Authors:** Maria das Dores Medina-Lopes, Luiz Augusto Casulari

**Affiliations:** ^1^The Secretariat of Health of the Federal District and the Ministry of Health, Brasília, DF, Brazil; ^2^The University Hospital of Brasília and Clinic of Endocrinology and Neurology (CLINEN), Brasília, DF, Brazil

## Abstract

Meningioma treatment includes observation of its growth or surgery with or without associated radiotherapy. However, drug treatment can be used for tumors deemed inoperable because of their size and location. Due to the presence of progesterone receptors, the use of antiprogestin mifepristone is recommended. This study describes a case of inoperable meningioma treated with mifepristone for 26 years without interruption. The patient is a 45-year-old woman diagnosed with plaque meningioma, extending from the bottom of her right orbit, through the length of the small wing of the sphenoid, part of the large wing of the sphenoid, especially near the superior orbital fissure, and at the ceiling of the orbit. As this meningioma was considered inoperable, treatment with 200 mg oral mifepristone was administered uninterruptedly for 26 years. This treatment initially halted the growth of the meningioma and subsequently resulted in a small reduction of its volume; however, the meningioma has persisted until the last evaluation. After five years of mifepristone use, hydroxyurea was added for nine months but was discontinued due to anemia and leucopenia. In conclusion, mifepristone was useful for the survival of the patient for those 26 years. The drug interfered with the natural history of the meningioma, which generally evolves to death in such long follow-up durations without associated surgery or radiation therapy.

## 1. Introduction

Meningiomas are classified into types I, II, or III. Type I meningiomas have very slow growth; however, rare variants (clear cell, chordal, papillary, and rhabdoid), as well as invasive or atypical grade II and type III anaplastic, are much aggressive. The specific chromosomal changes of these histological types account for their variable evolution [1].

Typically, type I meningioma does not require treatment, but only observation, in cases of growth that may require surgical intervention [2–5]. However, type II and type III meningiomas usually grow and require treatment. The suggested treatment strategies of these two types include maximum removal of the tumor that can be performed safely, active observation when the entire tumor has not been removed, and radiotherapy after partial tumor removal [6].

Several medical treatments have been developed for cases in which tumor growth cannot be controlled. These developments have occurred due to increased knowledge of specific molecular changes and the immune environment. Advances in molecular biology and genomics have led to new therapeutic modalities with promising results. Generally, therapies directed to hormones, cytotoxic chemotherapy, and molecular therapies with genomic analysis have been used (review, [7]).

Following the discovery of progesterone receptors on meningiomas [8–10], mifepristone has been of interest for the treatment of inoperable tumors or in cases for which surgery had no effect [11–17].

Mifepristone has been studied for many years for its potential therapeutic use as a potent antagonist of progesterone and glucocorticoids [18]. It is the first antisteroid with activity greater than or equal to that of the naturally occurring hormone agonist used clinically. It binds weakly to androgen receptors and does not bind to mineralocorticoid and estrogen receptors. Its use in the treatment of endometriosis, breast cancer, interruption of pregnancy, and contraception has been described, due to its antiprogestin activity [18]. Recently, its potential use in the treatment of vestibular schwannoma has been suggested [19]. Regarding the antiglucocorticoid effect of mifepristone, its use in depression, immunological abnormalities, inoperable adrenal cancer [18], tumors producing ectopic adrenocorticotropic hormone (ACTH) [20], and Cushing's disease [21] has been evaluated.

This article examined the antiprogestin action of mifepristone in the treatment of meningioma in a woman treated for 26 years without surgery and/or radiotherapy. For nine months, she also received hydroxyurea. To our knowledge, this case has the longest follow-up described in the literature.

## 2. Case Presentation

A white woman, 41 years of age, started having frequent headaches accompanied by extreme fatigue, nauseas, vomiting, and diplopia. She had a history of migraines since childhood. After successive consultations with several ophthalmologists, nothing was identified except diplopia. Exercises to correct the diplopia were prescribed without success.

At age 43, proptosis developed in her right eye; again, ophthalmological evaluations did not detect any abnormality. Therefore, the condition was considered a variation from normality. Subsequently, she observed that her right eye was on a lower plane in relation to her left eye and had altered motility, as if there were a lesion in the third cranial nerve. At that time, a cranioencephalic tomography revealed a pronounced thickening of the ceiling of the orbit and the small and large wings of the sphenoid; the upper rectus muscle was also lowered and thickened, especially posteriorly, suggesting meningioma in plaque, extending from the bottom of the right orbit, by the small wing of the sphenoid, in practically all its extension, and in part of the large wing of the sphenoid, especially along the superior orbital fissure, and at the ceiling of the orbit.

**Figure 1:**
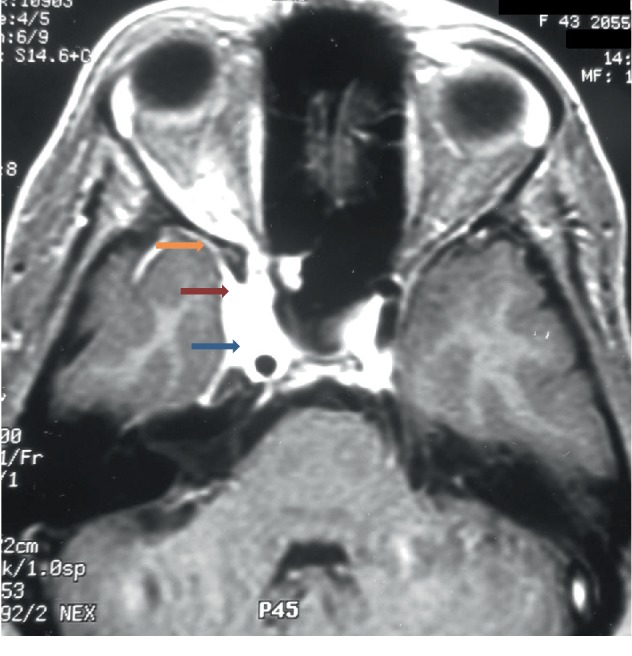
Axial sequence, after contrast, in T1, showing expansive, extra-axial, parasellar lesion, with intense enhancement after contrast infusion, involving the cavernous sinus and the carotid, without compromising their diameters (blue arrow); the lesion involves the apex of the orbit (red arrow), and there is thickening and enhancement of the dura mater (yellow arrow), suggestive of meningioma.

**Figure 2:**
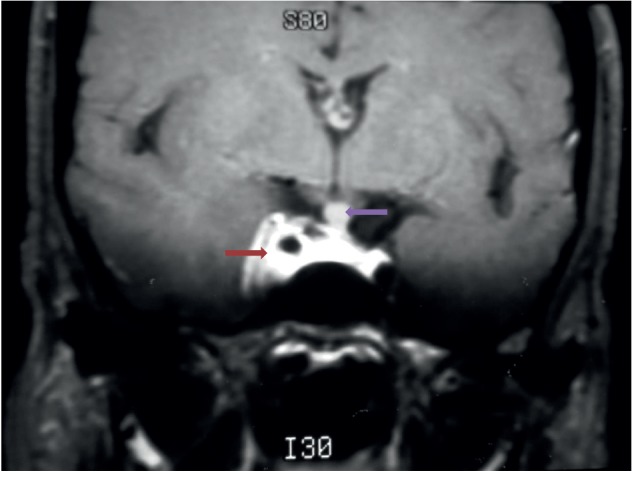
Coronal sequence, after contrast, in T1, showing expansive, extra-axial, parasellar lesion, with intense enhancement after contrast infusion, involving the right cavernous sinus and the carotid, without compromising their diameters (red arrow); thickening of the pituitary stem of an indeterminate nature (purple arrow).

**Figure 3:**
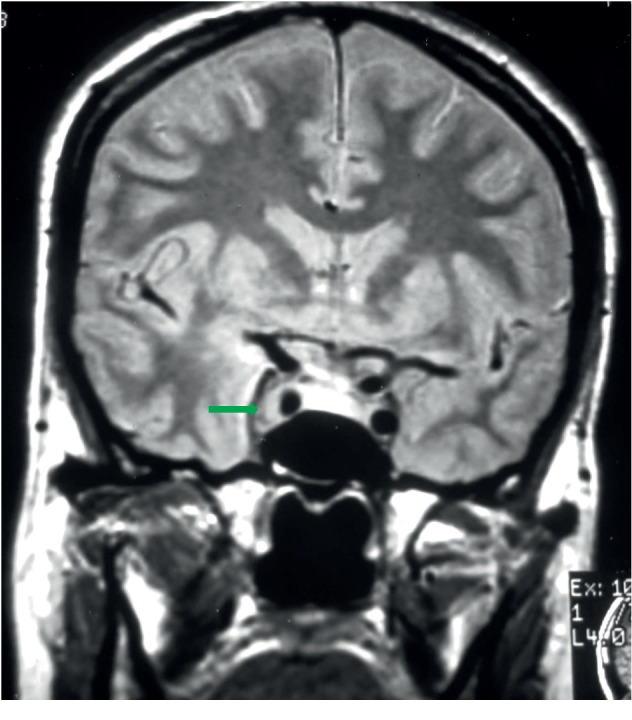
Coronal sequence, in T2, showing expansive, extra-axial, parasellar lesion, involving the right cavernous sinus and the carotid, without compromising their diameters (red arrow) with intermediate signal (green arrow).

Cranioencephalic magnetic resonance imaging (MRI) confirmed the diagnosis of meningioma (Figures 1–3) with the presence of a right parasellar lesion related to the cavernous sinus, previously involving superior orbital fissure impairment and later following the free edge of the tentorium. The lesion progressed to the suprasellar cistern, mainly to the right of the midline, apparently involving the pituitary infundibulum. These findings were highly suggestive of parasellar meningioma, with extension to the suprasellar cistern to the right of the midline and, later, to the free edge of the tentorium.

In her personal history, the patient had menarche at the age of 11, with later catamenias always regular; she was pregnant four times but had a spontaneous abortion in the first weeks of her second pregnancy. She had three children aged 23, 25, and 27 years from normal births. In the second full-term pregnancy, she had contractions from the seventh month of gestation and required rest until delivery.

**Figure 4:**
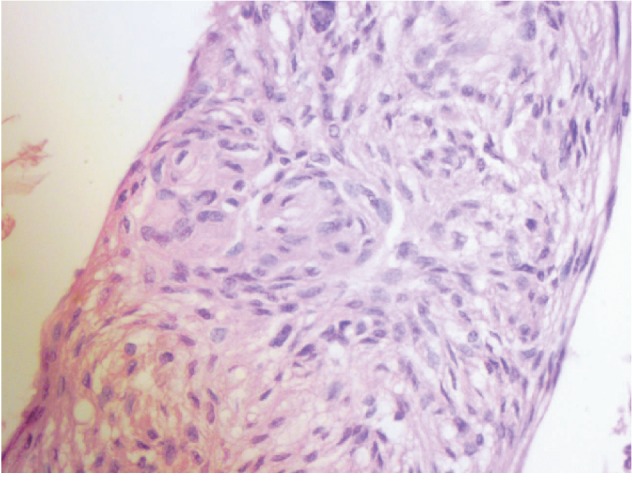
Neoplasia consisting of masses of elongated cells with imprecise limits, finely granular cytoplasm, and large rounded or oval nuclei; cytoplasmic invaginations configuring nuclear pseudoinclusions are visible at the top left, and the cells are arranged in lobes separated by fibrous tissue with some vorticilar aspects.

In the family history, her parents were immigrants to Brazil from Graciosa Island, in the archipelago of the Azores, and were not consanguineous. She was the eldest of six children. Her father died at 78 years of age due to metastatic prostate cancer. He was also a carrier of ankylosing spondylitis. Her uncle, five years younger than her father, had a history of seizures since adolescence; at the age of 55 years, he underwent surgery to remove a meningoteliomatous meningioma, as shown in Figure 4. Meningioma was found in the mother when she was 80 years of age.

The unanimous opinion of several neurosurgeons was that the patient had a tumor with no possibility of surgery.

She was started on 200 mg mifepristone daily, at age 44, as part of a research process being carried out by the Exelgyn Laboratory in France. She has uninterruptedly maintained this treatment to this day, for 26 years.

In the month following the diagnosis of meningioma, and before using mifepristone, her diplopia became pronounced and episodes of transient amaurosis appeared.  Table 1 shows the evolution of proptosis in the right eye and exotropia in the left eye before and during the 26 years of treatment. Significant worsening of both assessments was observed shortly after the tumor diagnosis was performed eight months before the start of mifepristone treatment. Relative stabilization of proptosis occurred after seven years. Exotropia, however, also worsened after diagnosis but with greater intensity than proptosis. She improved after four years of treatment and remained so until she was 66 years old. After 25 years of treatment, the motility of her eyeball was normal.

Other eye changes described were papilla edema observed during nine months of treatment with the drug, which disappeared after the fourth year.

The symptoms of headache, nausea, and vomiting were progressively decreased during the course of mifepristone treatment.

Five years after starting mifepristone treatment, oral hydroxyurea was initiated (500 mg three times daily). This treatment was continued for nine months and stopped due to major decreases in red and white blood cell counts. During the treatment, she had a 0.5 mm decrease in proptosis and wrinkles developed on the forehead. However, brain MRI showed no significant change in the tumor images.

**Figure 5:**
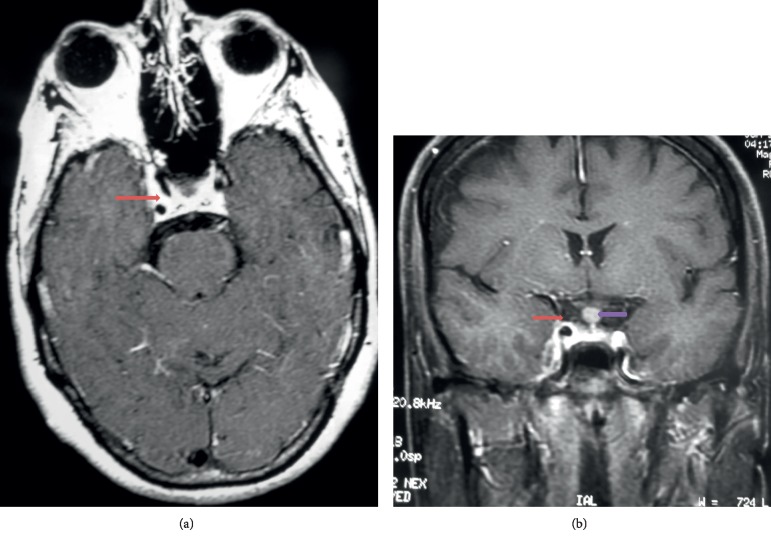
Magnetic resonance imaging after 23 years of mifepristone treatment. (a) Axial and (b) coronal sequences, in T1, after contrast, show significant reduction of the parasellar and dura mater expansive lesion (red arrow), with persisting thickening of the pituitary stem (purple arrow).

Serial MRI evaluations did not show significant changes in the images until 13 years of follow-up. However, a small decrease in tumor size was observed with 16 years of treatment (figures not shown). As shown in Figures 5(a) and 5(b), MRI at 23 years of treatment had a greater tumor decrease compared to the previous examination. The last MRI, after 26 years of treatment (figures not shown), showed no alteration in the tumor in relation to the evaluation at 23 years.

Table 2 shows the results of hormonal dosages before and during mifepristone use.

Four months before starting treatment with mifepristone, ACTH and cortisol concentrations were normal at 8-hour and 16-hour evaluations. However, after the start of the drug, both hormones increased above normal values and thus remained until the last evaluation after 26 years.

The patient's cycles were regular, and she should have menstruated four days after she started treatment with mifepristone, and also, the progesterone was 15 ng/mL; however, she never menstruated. Table 2 shows that the evaluations of gonadotropins (FSH and LH), estradiol, and progesterone were within the follicular phase of the menstrual cycle. After initiation of treatment with mifepristone, their FSH and LH levels remained above normal for the follicular phase for three months and then remained within the normal range. However, she started using 0.627 mg of conjugated estrogen which she continued for 14 years, concomitantly with mifepristone. During this period, FSH and LH remained suppressed.

Serial gynecological control showed that the endometrium was thickened. She underwent endometrial biopsies twice, showing cystic hyperplasia. After 29 months of using mifepristone, at age 47, she underwent a total hysterectomy. Histopathological examination showed uterine body leiomyomas, adenosis, and cystic endometrial hyperplasia, without cytological atypia.

Ultrasound monitoring of the patient's ovaries showed bilateral growth, with cystic cavities with septations. Bilateral oophorectomy was performed at age 57, after 12 years of mifepristone use. The histopathological diagnosis was follicular cysts and albicans bodies and serous cyst on the right and left ovary, respectively. After this procedure, her hormone levels were never compatible with menopause, even after 24 years of starting mifepristone treatment.

As shown in Table 2, about four years after the onset of the signs and symptoms of meningioma and one month before starting mifepristone treatment, the patient had thyroid-stimulating hormone (TSH) slightly above normal (6.8 *μ*UI/mL) and free T4 was below normal, but total T4 and total T3 had normal levels. Antithyroglobulin, antimicrosomal, and antiperoxidase antibodies were always undetectable. TSH levels increased four months after starting mifepristone (8.26 *μ*UI/mL) and remained at similar concentrations for up to 20 months; some levels of total T4 and free T4 during this same period were below the normal limit; however, total T3 levels were always normal. Replacement was started with 100 *μ*g L-thyroxine, and her TSH level remained below detectable concentrations. Her free T4 level was between 0.80 and 1.4 ng/mL until the last evaluation, 26 years after starting mifepristone therapy.

Before treatment, her prolactin levels were elevated, remained high (42.0 ng/mL) at two months, and normalized (17.0 ng/mL) at eight months after starting mifepristone. Until the last evaluation, 26 years after starting treatment, the levels remained normal (6.8 ng/mL).

The patient's IGF-1 level was always within normal limits for her age and sex, until the last evaluation, 26 years after the use of mifepristone. A growth hormone (GH) stimulus test to check for hormone deficiency was never performed.

Laboratory exams, such as complete blood count, lipidogram, liver and renal function tests, and levels of electrolytes, calcium, phosphorus, and parathyroid hormone (PTH), were within normal limits before the treatment was started and did not change during the 26 years of observation.

## 3. Discussion

We describe the case of a woman with inoperable meningioma treated using mifepristone without interruption for 26 years. The lack of tumor growth and its decrease may have been due to the chronic use of this drug; this possibility was evaluated by clinical improvements and radiological imaging.

Serial ophthalmological assessments suggested tumor growth within three years after diagnosis. The first ophthalmic signs of meningioma growth stabilization appeared after two years of mifepristone use. However, they were more remarkable after seven years, with improvement of proptosis and exotropia of the right eye. At 10 years of treatment, there was a marked improvement of these parameters in both eyes. At age 57, she underwent bilateral oophorectomy 12 years after starting mifepristone. After 16 years of mifepristone use and four years of surgical removal of the ovaries, there was an even greater improvement in ophthalmologic assessments.

Some characteristics of the patient were compared with those more commonly observed in patients with meningioma. For example, in a cohort of 12,384 patients diagnosed with meningioma, women (75%), white (79%), and married (53%) predominated [22]. An extensive case-control study [23] found that a family history of meningioma was associated with a higher frequency of this tumor. In the present case, the patient's paternal uncle and the mother had meningiomas.

Any comparison of the 26-year long-term survival with mifepristone with published results must consider the type of meningioma. This is because the survival of patients with type I meningioma is much greater than that of types II and III [2–5, 7]. As there was no histological study of the tumor, some of its characteristics in presentation led us to believe it to be a type II or atypical meningioma. The signs and symptoms included rapid tumor growth, which does not occur with type I, but with a much less intense evolution than those observed in type III. Type III has very rapid growth, with a 10-year survival rate of 14–34% [7]. In addition, the MRI images were suggestive of type II or atypical tumors.

In the follow-up of 244 untreated meningioma patients, 44% had growth during monitoring for 3.8 years. The factors associated with this evolution were age under 60 years, hyperintensity in MRI, absence of calcification, and a diameter greater than 26 mm [5]. All these factors were present in the current patient, which suggests that she would have had a worse prognosis if she had not been treated with mifepristone.

A mifepristone dose of 200 mg per day is most commonly used for the treatment of meningioma because it has significant antiprogesterone but less antiglucocorticoid activity [14]. The ingestion of doses greater than 100 mg results in constant serum concentrations. This is due to the saturation of alpha 1 acid glycoprotein, the serum protein to which mifepristone binds. This limits and regulates drug availability in tissues [24]. This is one of the justifications for not using dosages greater than 100 mg [24]. However, mifepristone exceeding that dose has been shown to be metabolized [25, 26] by demethylation and hydroxylation, resulting in monodemethylated, didemethylated, and hydroxylated metabolites [24, 27]. These metabolites retain considerable affinity for progesterone and glucocorticoid receptors [24, 27]. Serum concentrations of monodemethylated metabolite exceed those of mifepristone [25, 26]. When a dosage of 100 mg mifepristone is increased to 800 mg, serum concentrations remain the same, while concentrations of metabolites increase [27, 28]. Mifepristone has difficulty crossing the blood-brain barrier, but concentrations of demethylated metabolites were four times greater than those of mifepristone in the rat brain [27]. It is possible that the metabolites of mifepristone are more important for their antiprogestin and antiglucocorticoid effects than mifepristone [25, 26]. Thus, higher doses of mifepristone may be more useful in the successful treatment of intracranial tumors. However, this possibility needs to be demonstrated in further research.

Evaluation of the action of mifepristone on meningioma is difficult due to the low growth rate of these tumors. Thus, in addition to the imaging evaluation, cranial pair paralysis and ophthalmological evaluations for acuity and visual fields should also be objectively documented, along with neurological examinations [16]. To our knowledge, this is the first case in which these serial evaluations, along with imaging evaluations, were performed in the long term, with the use of mifepristone for the treatment of meningioma.

The first publication evaluating mifepristrone in the treatment of inoperable meningiomas at a dose of 200 mg per day, ranging from two to 31 months, observed objective improvements in tomography or resonance images of 5/14 treated patients [11]. In a subsequent publication with 28 patients, ranging from 2 to 157 months, they concluded that minor regression of meningioma that can result in significant clinical benefit is suggested in the male and premenopausal female subgroups of patients [14].

More recently, Touat et al. [15] treated three women with multiple meningiomas with mifepristone, with follow-up time duration ranging from five to nine years. Radiologically, they observed a decrease in two patients and stabilization in the third. Long-term treatment with mifepristone has been described for up to 13 years in one patient and 10 to 12 years in six patients [13]. The patient described here was followed for 26 years, a long time not yet described in the literature.

Although mifepristone is well-tolerated, a systematic literature review found no evidence to recommend its use in inoperable meningiomas [16].

The only randomized placebo study showed no significantly greater effect of mifepristone. The multicenter, prospective, randomized, placebo-controlled, phase III research study assessed 80 patients who received mifepristone and 84 who received a placebo. Twenty-four patients (30%) completed two years of mifepristone use without disease progression, adverse effects, or other reasons to discontinue treatment. The 28 (33%) patients administered that the placebo also completed the study. No statistical difference was observed between the two groups in relation to failure-free or overall survival [17]. The authors did not reach a conclusion because 88% of the meningiomas in the sample had progesterone receptors but did not respond to treatment with mifepristone. Nevertheless, they speculated that meningiomas without progesterone receptors have a higher likelihood of recidivism than those with such receptors [29]. Thus, the latter would be less responsive to mifepristone and would see meningioma progression and those with a good response would be progesterone receptor-positive. An interesting suggestion for the nonaction of mifepristone in progesterone receptor-positive meningiomas may be linked to the presence of an estrogen receptor. The anticancer action of progesterone in hormonally sensitive tumors usually occurs through the downregulation of the estrogen receptor, which is present in less than 5% of meningiomas [17].

Soon after beginning mifepristone treatment, the patient experienced amenorrhea, which persisted for the entire follow-up period. In the hormonal evaluations, FSH and LH levels increased in the first three months of mifepristone use but later remained within the range of the follicular phase of the cycle, which is the lowest during the menstrual cycle. These gonadotropin behaviors were also observed in treatment with mifepristone [12, 30]. Decreases in LH and FSH secretion in normal women during the menstrual cycle, with the use of mifepristone, have been described [31, 32]. This behavior of gonadotropins may be due to the negative feedback effect of increased testosterone, estradiol, and estrone caused by mifepristone [12]. In a randomized study, the 14 premenopausal patients who used mifepristone had menstrual cycle alteration [17].

Serial evaluations showed cystic endometrial hyperplasia. After two years, the patient underwent a total hysterectomy, following which the histopathological examination showed leiomyomas of the uterus body, adenosis, and cystic endometrial hyperplasia, without cytological atypia. Endometrial hyperplasia with the chronic use of mifepristone for the treatment of meningioma [13, 14, 17] and in Cushing's disease [21] has been described. Endometrial polyps due to the blockage of the contraregulatory action of estradiol progesterone have also been described [30]. In a case-control study [23], women with meningiomas were more likely to develop hormone-related diseases such as uterine leiomyoma, as observed in the present case.

Twelve years after starting mifepristone, at 57 years of age, she presented bilateral growths on her ovaries. She underwent bilateral oophorectomy, and serous cystadenoma was found in her left ovary. This association was also reported in one of the three patients treated with mifepristone for multiple meningioma [15].

The patient developed subclinical hypothyroidism soon after starting mifepristone, which has also been described previously [13]. The mechanism may be linked to mifepristone interference in the hypothalamic-pituitary-adrenal axis. Excess cortisol in Cushing's syndrome is associated with decreased TSH level; the action of mifepristone may block this effect on TSH [33]. In the randomized study, no hypothyroidism was observed in either the mifepristone or placebo group [17].

However, it is more likely that the patient developed hypothyroidism secondary to the presence of the meningioma in the region near the hypothalamus and hypophysis. Patients with lesions in this region may secrete TSH that is biologically inactive, although it is immunologically active and recognized by the antibody used in the assay of its dosage. In this case, the control of hormone replacement with L-thyroxine is with the dosage of free T4, kept above 1.0 ng/mL. Unlike primary hypothyroidism, in which TSH plays an important role in hypothyroidism secondary to hypothalamic injury, the levels may be normal or slightly elevated. With L-thyroxine replacement, its secretion is quickly suppressed [34, 35].

The patient was treated with hydroxyurea for nine months. There was a decrease in proptosis, and she began to develop wrinkles on her forehead. However, MRI assessment showed no alterations in the tumor. Due to anemia and leukopenia, we opted not to continue this treatment. A first publication that reported the use of hydroxyurea for the treatment of meningioma had very good results: three patients had tumor reductions of 74%, 60%, and 15%, respectively, and a fourth patient with type III meningioma had no tumor recurrence two years after surgery [36]. Retrospective and prospective studies showed that hydroxyurea had an initial beneficial effect, followed by disease progression (review, [7]). However, hydroxyurea in association with imatinib [37, 38] and verapamil [39] presented promising results.

In conclusion, ophthalmological serial evaluations showed that the tumor grew in the first three years, with progressive worsening of exotropia and ocular proptosis. If mifepristone had not been started, most probably, the patient would have developed a loss of vision and even died within a few years, as described for grade II meningiomas. Given the natural history of type II meningiomas, with or without treatment, it is unlikely that the patient would have survived for 26 years if she had not been receiving mifepristone.

## Figures and Tables

**Table 1:** Evolution of right eye proptosis and left eye exotropia before and during treatment with mifepristone.

	Exophthalmometry (mm)	Exotropia (diopters)
*Before mifepristone (month)*		
8	3	8
4	5	12
*After mifepristone (year)*		
0.2	6	18
2	6.5	18
4	7	—
5	6	8
6	6.5	8
7	6.5	7
9	5.5	—
10	5.5	8
16	5.0	6
25	Normal	Normal

**Table 2:** Hormonal dosages before and during mifepristone use.

Hormone^∗^	Basal	After mifepristone	
Cortisol 8 h (NV 4.3–22.4 ng/mL)	6.0	239	
Cortisol 16 h (NV 2–15 ng/dL)	3.0	37	
ACTH 8 h (NV up to 160 pg/mL)	90	420	
ACTH 16 h (NV up to 50 pg/mL)	110	85	
FSH (NV follicular phase 2.5–10.2 mIU/mL)	9.4	0,1^†^	8.3^ǂ^
LH (NV follicular phase 2.5–12 mIU/ml)	6.75	0,8^†^	6.77^ǂ^
Estradiol (NV 19.5–144 pg/mL)	151	—	35.8^ǂ^
Progesterone (NV follicular phase < 1.4 ng/mL)	0.22	—	
TSH (NV 0.6–4.5 mcUI/mL)	6.8	<0.01^§^	
T4 total (NV 5.2–12,7 mcg/dL)	6.47	—	
T4 free (NV 0.72–1.72 ng/mL)	0.68	1.38^§^	
T3 total (NV 60–181 ng/dL)	92	—	
Prolactin (NV up to 26 ng/mL)	76.8	17.0	
IGF-1 (37–219 ng/mL)	90.6	91	

^∗^ACTH = adrenocorticotropic hormone; FSH = follicle-stimulating hormone; LH = luteinizing hormone; TSH = thyroid-stimulating hormone; IGF-1 = insulin growth factor 1. ^†^Use of 0.625 mg equine-conjugated estrogen; ^ǂ^after bilateral oophorectomy performed at age 57; ^§^use of 100 mcg L-thyroxine.
